# Supplementary material for: The role of references and the elusive nature of the chemical bond
Source: Nat Commun. 2022 Jun 9;13:3327. doi: 10.1038/s41467-022-31036-6 (PMC9184482; doi:10.1038/s41467-022-31036-6)
Supplement: Supplementary file 1 — Supplementary Information [file 41467_2022_31036_MOESM1_ESM.pdf]

Supplementary Information: The role of references and the elusive nature of the chemical bond. A. Martín Pendás et al.

Supplementary Note 1. The standard model of the chemical bond in H_2^+ and H_2

All electronic structure calculations have been done using the `GAMESS US` code.[1]

A standard atomic reference is taken as the H atom and its exact one-electron states ϕ_{nl}, $E_{nl} = -1/(2n^2)$ au. This reference is chosen since at large interatomic distance R the energy of the system tends to that of a free H atom (*vide infra* for further comments on this). Now, the ground and first excited state molecular wavefunctions $\Psi_{g,u}$ can be recast as $\Psi_{g,u} = N_{g,u}(\phi_a \pm \phi_b)$, where $\phi_{a,b}$ are symmetric atomic orbitals centered at both nuclei, and $N_{g,u} = 1/\sqrt{2 \pm 2S_{ab}}$, $S_{ab} = \langle \phi_a | \phi_b \rangle$. This can be done in many ways. Ruedenberg and coworkers' procedure, based on a singular value decomposition, yields the so-called quasi atomic orbitals (QUAOs). With this QUAO *internal reference* we can now: (i) compare the distortion of the QUAOs with respect to the ϕ_{1s} orbital. This distortion can be further decomposed into a spherical contraction/expansion and a non-spherical polarization. (ii) Decompose both the total E, T, V energies as well as the total electron density $\rho = \Psi^2$ into QUAO related quantities. Squaring the Ψ amplitude leads to the sum of the squares of the quasi atomic densities and to an *interference* term: $\rho = \rho_a \rho_b + \rho_I$, where $\rho_a = \phi_a^2$, and $\rho_b = \phi_b^2$. Similarly, an straightforward decomposition of the energy E allows to write both the potential and the kinetic energies of the ground state as a sum of intra-atomic and interatomic contributions: $V = V_{intra} + V_{inter}$; $T = T_{intra} + T_{inter}$. The intra-atomic potential V_{intra} measures the attraction of an electron in any of the two symmetric QUAOs and its associated nucleus, while $V_{inter} = V_{qc} + V_I$ contains the *quasiclassical* attraction of the electron in a QUAO with the other nucleus and the internuclear repulsion, V_{qc}, and an *interference* term V_I coming from the electron-nucleus attraction of the ρ_I. Similarly, T_{intra} is the kinetic energy of an electron in a QUAO, while $T_{inter} = T_I$ is entirely due to interference. This leads to the, let us say, *standard model* of bond formation: the QUAO passes from slight expansion at large R to significant contraction at equilibrium; $E_{intra} = T_{intra} + V_{intra}$ is destabilizing, so it is E_{inter} which drives bonding. V_{qc} is only slightly stabilizing due to QUAO interpenetration, but V_I is destabilizing, so that it is the T_I lowering which drives the system to an equilibrium geometry. It is also easily shown that the accumulation of density in the internuclear region is entirely caused by interference, driven by expansion of QUAOs at large distances and by contraction at shorter ones. As R decreases and T_{intra} starts dominating, orbital contraction guarantees the restoration of the virial theorem. Many researchers have contributed to the details of this image over the years.

Supplementary Note 2. A primer on Real Space Chemical Bonding

Real space reasoning uses proper quantum mechanical observables to construct orbital invariant descriptors with chemical meaning. This is done by using quantum mechanical objects which are already orbital invariant, like reduced densities and reduced density matrices (RDs, RDMs). An identification of spatial regions with chemical concepts is also necessary. This is usually done through spatial partitionings, normally induced by the topology of a scalar field. For instance, the topology of the electron density, ρ, induces an atomic partitioning which has been used in the Quantum Theory of Atoms in Molecules (QTAIM) explored by R. F. W. Bader and coworkers.[2] Similarly, the topology of Becke and Edgecombe's[3] electron localization function (ELF) provides a partition of the space into cores, lone pairs and bonding domains, etc.

Once atoms (or electron-pair domains) are available, chemical bonding descriptors are built. Both the electron-counting perspective (leading to populations and bond orders) as well as the energetic view that provides bond strengths are needed. These are offered by, for instance, electron distribution functions (EDFs) and the interacting quantum atoms approach (IQA).

The energetic face of bonding: Interacting Quantum Atoms (IQA)

Given an atomic spatial partitioning, the interacting quantum atoms (IQA) energy partition considers the one- and two-domain division of the non-relativistic Born-Oppenheimer electronic energy[4] described in the following equation,

$$\begin{aligned} E &= \sum_{\mathrm{A}} E_{\mathrm{self}}^{\mathrm{A}} + \sum_{\mathrm{A>B}} E_{\mathrm{int}}^{\mathrm{AB}} \\ &= \sum_{\mathrm{A}} T^{\mathrm{A}} + V_{\mathrm{ne}}^{\mathrm{AA}} + V_{\mathrm{ee}}^{\mathrm{AA}} + \sum_{\mathrm{A>B}} V_{\mathrm{nn}}^{\mathrm{AB}} + V_{\mathrm{ne}}^{\mathrm{AB}} + V_{\mathrm{ne}}^{\mathrm{BA}} + V_{\mathrm{ee}}^{\mathrm{AB}}, \end{aligned} \tag{1}$$

wherein $E_{\mathrm{self}}^{\mathrm{A}}$ and $E_{\mathrm{int}}^{\mathrm{AB}}$ are the IQA self and interaction energies of atom A and pair AB, while T^{A} denotes the kinetic energy of atom A. Finally, the terms $V_{\mathrm{ne}}^{\mathrm{AB}}$ and $V_{\mathrm{ee}}^{\mathrm{AB}}$ stand for (i) the attraction between the nucleus of domain A and the electrons of atom B and (ii) the repulsion between the electrons in atom A with those in basin B, respectively. The self-energy of an atom is the trace of its *in vacuo* Hamiltonian over the atomic region it occupies in a molecule. In a process where the atoms of a system dissociate their self-energies tend to the free atomic energies.

We can get further insight about the nature of the interaction between two atoms by separating the electronic repulsion into its Coulombic and exchange-correlation components. This splitting allows, in turn, the separation of the IQA interaction energy of a pair AB as[4]

$$E_{\mathrm{int}}^{\mathrm{AB}} = V_{\mathrm{cl}}^{\mathrm{AB}} + V_{\mathrm{xc}}^{\mathrm{AB}} = E_{\mathrm{ion}}^{\mathrm{AB}} + E_{\mathrm{cov}}^{\mathrm{AB}}. \tag{2}$$

Usually binding is measured relative to appropriate reference for the quantum fragments A, with $E^{A,0}$ energies. Then $E_{self}^{A} - E^{A,0} = E_{def}^{A}$ is called the atomic or fragment deformation energy, which corresponds to a combination of the traditional promotion energy and other effects, like spin-recoupling, true electronic deformation, etc.[5] We have shown that the IQA interaction energies behave as *in situ* bond energies. IQA thus provides an invariant decomposition of the energy into group deformations and bond contributions in which covalent and ionic energies acquire rather pure forms.

All IQA calculations have been performed with the `PROMOLDEN CODE`.[6]

Broken symmetry IQA in H_2^+

We refer to Fig. 1 in the main text and build two normalized densities (formally one-particle density matrices) integrating to one for each of the two possible electron distributions: $\tilde{\rho}^a$ for the $(1,0)$ and $\tilde{\rho}^b$ for $(0,1)$ distributions. In this one electron case this is immediate and, for instance, $\tilde{\rho}^a(\mathbf{r}) = 2\rho(\mathbf{r})w_a(\mathbf{r})$, where w_a (the basin indicator function) is 1 if $\mathbf{r} \in a$ and zero otherwise. The system is now visualized as an H^a atom interacting with a proton at H^b or vice versa. Since the system is symmetric,the Figue is also valid. Consider for instance an electron confined in the left H_a region, described by the previous $\tilde{\rho}_a$ density. Its self-energy is $\tilde{T}_a + \tilde{V}_{en}^{aa} = 2E_{self}^a = E_{self}^a + E_{self}^b$. It is equally found that $\tilde{E}_{int}^{ab} = E_{int}^{ab}$, being safely identified with the classical interaction of a proton and an atom described by a $\tilde{\rho}^a$ density, dominated by an R^{-2} charge-dipole tail, and that $\tilde{V}_{en}^{aa} = V_{en'}$ in Fig. 1.

IQA in H_2

The self-energy of each atom (e.g. H_a) contains the kinetic energy, T^a, of the electrons in a (no interference whatsoever), the electron-own nucleus attraction V_{en}^{aa} and a new intra-atomic electron repulsion, V_{ee}^{aa} term, which plays a similar role to V_{sc}, although the latter is assigned to an interatomic contribution. E_{int}^{ab} includes also the interatomic electron repulsion V_{ee}^{ab}. As with E_{intra}, E_{self}^a is repulsive, so that all binding comes from E_{int}^{ab}, which can be partitioned into the classical component $V_{cl}^{ab} = \int_a d\mathbf{r}_1 \int_b d\mathbf{r}_2 \, \rho_t(\mathbf{r}_1)\rho_t(\mathbf{r}_1)/r_{12}$, where the total electronic plus nuclear density ρ_t is used, and the exchange-correlation energy V_{xc}^{ab}. The first is the all-classical interaction of the system of electrons and nuclei considered as classical particles with the quantum-mechanically determined distribution, whereas the second measures the quantum-mechanical deviation to this interaction. In H_2, the only stabilizing contribution is V_{xc}. If two interacting moieties a, b carry net charges Q_a, Q_b, V_{cl}^{ab} will be dominated by a monopolar Q_aQ_b/R_{ab} Coulombic contribution. Similarly, V_{xc} is dominated by a $-\delta^{ab}/(2R_{ab})$ term, where δ^{ab} is the a, b covalent bond order.[7]

The electron-counting face of bonding: Electron Distribution Functions

Electron counting provides access to the more qualitative view of chemical bonding in which the number of electrons engaged in sharing or in pure transfer between atoms gives rise to bonding descriptors like bond orders. In real space we simply examine the distribution of the electron population in the atomic regions in which we have divided the space.

EDFs are defined as follow. Given an N–electron molecule and an exhaustive partition of the real space ($\mathcal{R}^3$) into m arbitrary regions $\Omega_1, \Omega_2, \ldots, \Omega_m$ ($\Omega_1 \cup \Omega_2 \cup \cdots \cup \Omega_m = \mathcal{R}^3$), an EDF is the distribution function formed by all the probabilities $p(n_1, n_2, \ldots, n_m)$ of finding exactly n_1 electrons in Ω_1, n_2 electrons in Ω_2, $\ldots$, and n_m electrons in Ω_m, $\{n_p\}$ being integers ($n_i \in \mathcal{N}$) satisfying $n_1 + n_2 + \cdots + n_m = N$. This view is in accord with considering subsystems as open quantum systems in which number operators do not commute with the subsystem hamiltonian. In this way, Ψ is not an eigenstate of the operator defining the number of electrons in domain Ω_i, $\hat{N}_{\Omega_i}$. This means that the average number of electrons in Ω_i is not an eigenvalue of $\hat{N}_{\Omega_i}$, so that measuring the number of electrons in the domain will render values n_{Ω_i} ranging from 0 to N, the total number of electrons, with a defined set of probabilities, $p(n_{\Omega_1})$. This is the one-fragment EDF for domain Ω_i. To obtain these probabilities or, in general, the multivariate electron distribution functions $p(n_1, n_2, \cdots, n_m)$, one needs $\Psi(1, \ldots, N)$, Ψ being the complete wave function,

$$p(n_1, n_2, \ldots, n_m) = N!\Lambda \int_D \Psi^\star \Psi d\mathbf{x}_1 \cdots d\mathbf{x}_N, \quad (3)$$

where D is a multidimensional domain in which the first n_1 electrons are integrated over Ω_1, the second n_2 electrons over Ω_2, $\cdots$, and the last n_m electrons over Ω_m, and $N!\Lambda =$

$N!/(n_1!n_2!\cdots n_m!)$ is a combinatorial factor that accounts for electron indistinguishability. The 3D domains of these integrations can be arbitrary, but when using QTAIM atomic basins, a partition of the N electrons of the molecule that assigns a given number of electrons (including possibly 0) to each of these regions is called a *real space resonance structure* (RSRS)[8] and there are $N_S = (N+m-1)!/[N!(m-1)!]$ of these for a given N,m pair. With the notation $S(n_1, n_2, \ldots, n_m) \equiv S(\{n_p\})$, or simply $(n_1, n_2, \ldots, n_m) \equiv \{n_p\}$, we label the resonance structure having n_1 electrons in Ω_1, n_2 electrons in Ω_2, ..., and n_m electrons in Ω_m. If electrons are spin-seggregated, then we come to spin-resolved EDFs, and a set of probabilities $p(n_1^\alpha, n_1^\beta, n_2^\alpha, n_2^\beta, \ldots, n_m^\alpha, n_m^\beta)$ which gives extremely fine-grained information about how electrons and their spins distribute.[9]

The computation of $p(n_1, n_2, \ldots, n_m)$ for all the RSRSs provides all the statistical moments of the electron populations, including the average number of electrons in a given region, or its fluctuation. The average population of region Ω_i is obviously given by

$$N_i = \langle n_i \rangle = \sum_{\{n_p\}} n_i \times p(\{n_p\}) = \sum_{n_i} n_i p_i(n_i). \tag{4}$$

It is not difficult to show that the number of shared pairs between two regions may be obtained directly by counting the number of intra- and interpairs.[10] This has given rise to the so-called localization and delocalization indices, $(\lambda^{ii}, \delta^{ij})$, which determine the number of *localized* and *delocalized* pairs. The latter, which is the covalent bond-order in real space can be obtained from the $p(\{n_p\})$ probabilities as

$$\delta^{ij} = -2\text{cov}(i,j) = -2\left[\langle n_i n_j\rangle - \langle n_i\rangle\langle n_j\rangle\right] = \tag{5}$$

$$-2\left[\sum_{\{n_p\}} n_i n_j \times p(\{n_p\}) - \langle n_i\rangle\langle n_j\rangle\right] = \tag{6}$$

$$-2\sum_{n_i n_j}(n_i - N_i)(n_j - N_j)p(n_i, n_j) = 2N_{ij} \tag{7}$$

where the -2 factor has been included to comply with the usual definition of δ in terms of the exchange-correlation density and to ensure that the bond order for an ideal single bond is equal to 1,

$$\delta^{ij} = -2\int_{\Omega_i}\int_{\Omega_j} d\mathbf{1}\; d\mathbf{2}\rho_{xc}(1,2). \tag{8}$$

The localization index is given by

$$\lambda_{ii} = N_i - \text{cov}(i,i) = N_i - var(i) = N_i - \sum_{n_i}(n_i - N_i)^2 p(n_i) = N_{ii} \tag{9}$$

From equations 5-9 it is clear that $N_{ii} = N_i$ if the variance is zero and that $N_{ij} = 0$ if the covariance is zero. This is the starting point for a complete theory of chemical bonding based on the fluctuation of electron populations. There is chemical bonding between two regions if their electron populations are not statistically independent. A sum rule, that classifies electrons into localized and delocalized sets appears:

$$N = \sum_{\Omega_i} N_i = \sum_{\Omega_i} \lambda^{ii} + \frac{1}{2}\sum_{\Omega_i \neq \Omega_j} \delta^{ij}. \tag{10}$$

Suitable generalizations in the case of multi-center bonding exist.[11]

Ionic and covalent structures in 2c-2e bonds

The statistical link between the fluctuation of electron populations and the standard energetic and bond order descriptors allows to map all coarse-grained (i.e. condensed at the atomic level) possible $(2c-2e)$ bonds through simple models. In a two-center, two-electron system there are only three RSRSs: $(2,0)$, $(1,1)$, $(0,2)$, where we label how many electrons lie in each of the a,b domains. The central structure is obviously identified with the valence-bond covalent structure, while the other two describe ionic distributions. The EDF space is two-dimensional, since $p(2,0)+p(1,1)+p(0,2)=1$, and all bond indices become fully mapped in this 2D space. A convenient coordinate system can be built with the probability that any of the electrons lie in one of the basins, e.g. the left one, which we call p and provides a measure of heteropolarity, and a correlation factor $-1 \leq f \leq 1$ that determines how the electronic motion is correlated. $f=1$ means that an electron is completely excluded from one domain if the other is already in it (positive correlation) and $f=-1$ implies that the two electrons are always found together within the same domain (negative correlation). The correlation factor here defined plays the same role as that used in density matrix theory, where $\rho^2(r_1,r_2)=\rho(r_1)\rho(r_2)(1-f)$. The (p,f) pair describes fully a 2c,2e link at this level: $p(2,0)=p^2-p(1-p)f$, $p(1,1)=2p(1-p)(1+f)$ and $p(0,2)=(1-p)^2-p(1-p)f$.[12] Obviously, for two symmetric a,b domains, $p=1/2$, so that $p(2,0)=p(0,2)=1/4(1-f)$, and $p(1,1)=1/2(1+f)$. When $f=0$, as in a single determinant description where the opposite spin electrons are statistically independent, $p(2,0)=p(0,2)=1/4$, $p(1,1)=1/2)$, a binomial distribution. Positive f factors lead to a decrease of $p(2,0)=p(0,2)$ coupled to an increase of $p(1,1)$. Electron correlation localizes the electrons in the basins.

Positive correlations between the electrons can be also described through quasi-distinguishable electrons, which have different probabilities of residing in the two centers. Let us consider two quasi-distinguishable, statistically independent electrons, the first having a $p(1,0)=p_a=1-p(0,1)$ distribution and the second exhibiting a $p(0,1)=p_b=1-p(1,0)$ one. Here we expect that p_a and p_b are large, so that the electrons are more or less delocalized over basins a and b, respectively. Their statistical two-electron distribution is the direct product of both, leading to $p(2,0)=p_a(1-p_b)$, $p(0,2)=p_b(1-p_a)$, and $p(1,1)=p_ap_b+(1-p_a)(1-p_b)$. Notice that for a symmetrical system, like H_2, $p_a=p_b$, and there is a one-parameter family of solutions which are equivalent to those with $p=1/2$, $f\geq 0$. This description is useful when considering the 2c,2e link as two independent 2c,1e bonds.

If we use these p,f parameters, the covalent bond order becomes $\delta=4p(1-p)(1-f)$. An ionic bond order $\iota=-Q_aQ_b$ where Q is the net charge of a center has also been defined.[13] In standard weakly correlated bonds with positive $f\sim 0$, the EDF is close to binomial, and δ peaks at $\delta=1$ for a purely covalent homopolar link with $p=1/2$. As electron correlation, f , or polarity, p, increases δ decreases. Moreover, for non-correlated links with $f=0$ $\iota=1-\delta$ so, in agreement with standard wisdom, the ionic and covalent bond orders are inversely correlated.

When f deviates from zero, the model describes positively or negatively correlated bonds. The latter case implies a bosonization of the link. Electrons try to delocalize together, giving rise to very large fluctuations. The most extreme 2c,2e case with $\delta=2$ occurs when $p(0,2)=p(2,0)=1/2$ and $p(1,1)=0$, i.e. when there is a resonance between the two non-orthogonal valence bond (NOVB) ionic structures. Thus, $f<0$ serves to separate cleanly, in real space, large fluctuations from the standard bonding regime.

Several rigorous bond-energy bond-order (BEBO)[13] relations can be uncovered using these real space descriptors. Under the IQA perspective a multipolar expansions shows that the first order ionic and covalent energies are immediately related to their corresponding bond orders. For an interaction between atoms A and B,

$$E_{ion}^{AB}\sim -\frac{\iota^{AB}}{R_{AB}} \quad E_{cov}^{AB}\sim -\frac{1}{2}\frac{\delta^{AB}}{R_{AB}}. \tag{11}$$

Notice that the arguments in the main text show that in the absence of ionic contributions (delocalization) a system is unbound. This means, for instance, that the good behavior of the conventional covalent Heitler-London function of H_2 is due to the large overlap of the H 1s functions, that introduce a considerable amount of *real space ionic* terms.

Natural Adaptive Orbitals

The covalent bond order measured by the delocalization index can be partitioned into exchange-channel contributions in an invariant way. To do that, the RDMs themselves are coarse-grained and diagonalized, so that a set of one-electron functions adding to the total n-center delocalization index can be obtained.[11] The procedure is relatively simple. In order to consider only the population fluctuations that lie behind electron delocalization, the n-th order cumulant densities (CDs), $\rho_n^c(\mathbf{r}_1, \ldots, \mathbf{r}_n)$ are introduced. These objects integrate to the total number of electrons N. A partition in centers of $\rho_c^1 = \rho$ provides the standard QTAIM atomic populations, that of $\rho_2^c = \rho_{\rm xc}$ gives rise to the well-known localization and delocalization indices which are real space covalent bond orders, and, in general, a decomposition of ρ_n^c reveals n-center bond orders. The CDs can be partially coarse-grained, leaving one electron coordinate free of the integration over centers, a process that describes a real space natural density of n-center bonding. Diagonalizing these densities we get sets of effective one electron functions, the natural adaptive orbitals (NAdOs),[14] together with their associated natural adaptive occupations. The latter decompose the electron population, the two-center, three-center, etc. bond orders into one-electron components as the order of the cumulant that is diagonalized increases. For instance, in order to partition the DI into one-electron terms, the third-order cumulant is integrated over two basins, A, B, and the resulting density is diagonalized. The sum of the occupation numbers of these orbitals add to the total delocalization index.

All distribution function calculations have been performed with the `EDF` code.[15]

Supplementary Note 3. H_2^+ IQA data

FCI/cc-pVTZ data in a.u. IQA atomic $T^A, E^A_{self}, E^{AB}_{int}, V^{AB}_{xc}, V^{AB}_{cl}, Q^A, \lambda^A, \delta^{AB}, V_{en}$ values.

#R/2	KinA	SelfA	Interaction	XC	Classic	QA	LocA	DI	Ven
0.3	0.639984	-0.286853	0.572038	-0.212492	0.784530	0.499997	0.250003	0.500006	-0.926837
0.4	0.553640	-0.305720	0.308549	-0.195100	0.503649	0.499982	0.250018	0.500036	-0.859361
0.5	0.484773	-0.313561	0.176198	-0.179639	0.355837	0.499979	0.250021	0.500042	-0.798334
0.6	0.430106	-0.315236	0.102085	-0.166082	0.268167	0.499982	0.250018	0.500037	-0.745342
0.7	0.386544	-0.313531	0.057633	-0.154126	0.211759	0.500028	0.249972	0.499944	-0.700075
0.8	0.351717	-0.310063	0.029587	-0.143597	0.173184	0.500009	0.249991	0.499982	-0.661779
0.9	0.323694	-0.305678	0.011492	-0.134203	0.145695	0.500018	0.249982	0.499965	-0.629373
1.0	0.301050	-0.300894	-0.000342	-0.125733	0.125391	0.500038	0.249962	0.499923	-0.601944
1.1	0.282647	-0.296098	-0.008142	-0.118056	0.109914	0.500043	0.249957	0.499915	-0.578745
1.2	0.267725	-0.291459	-0.013327	-0.111123	0.097796	0.499915	0.250085	0.500170	-0.559185
1.3	0.255566	-0.286984	-0.016310	-0.104628	0.088318	0.499994	0.250006	0.500012	-0.542549
1.4	0.245802	-0.282847	-0.018064	-0.098685	0.080621	0.499966	0.250034	0.500069	-0.528649
1.5	0.238005	-0.279045	-0.018766	-0.093121	0.074355	0.500001	0.249999	0.499998	-0.517050
1.6	0.231947	-0.275555	-0.018761	-0.087943	0.069182	0.499980	0.250020	0.500039	-0.507502
1.7	0.227308	-0.272399	-0.018134	-0.083037	0.064903	0.500042	0.249958	0.499917	-0.499707
1.8	0.223925	-0.269603	-0.017169	-0.078463	0.061294	0.500048	0.249952	0.499903	-0.493527
1.9	0.221638	-0.267106	-0.015902	-0.074141	0.058239	0.500067	0.249933	0.499866	-0.488744
2.0	0.220320	-0.264921	-0.014541	-0.070133	0.055592	0.499982	0.250018	0.500037	-0.485241
2.2	0.219928	-0.261283	-0.011531	-0.062838	0.051307	0.499943	0.250057	0.500114	-0.481211
2.4	0.221681	-0.258428	-0.008716	-0.056608	0.047892	0.500012	0.249988	0.499975	-0.480108
2.6	0.224700	-0.256221	-0.006532	-0.051448	0.044916	0.500010	0.249990	0.499979	-0.480921
2.8	0.228300	-0.254526	-0.004905	-0.047159	0.042254	0.499993	0.250007	0.500014	-0.482827
2.9	0.230159	-0.253841	-0.004254	-0.045284	0.041030	0.499986	0.250014	0.500027	-0.484000
3.5	0.239894	-0.251320	-0.001794	-0.036627	0.034833	0.500001	0.249999	0.499997	-0.491214
4.0	0.244911	-0.250471	-0.000881	-0.031698	0.030817	0.499991	0.250009	0.500017	-0.495381
4.5	0.247591	-0.250113	-0.000445	-0.028004	0.027559	0.499998	0.250002	0.500004	-0.497704
5.0	0.248892	-0.249971	-0.000242	-0.025122	0.024880	0.499999	0.250001	0.500002	-0.498863

Supplementary Figure 1: He_2^{2+} cc-pVTZ/FCI energy curve. The Coulombic repulsion of two unit point charges is superimposed in green.

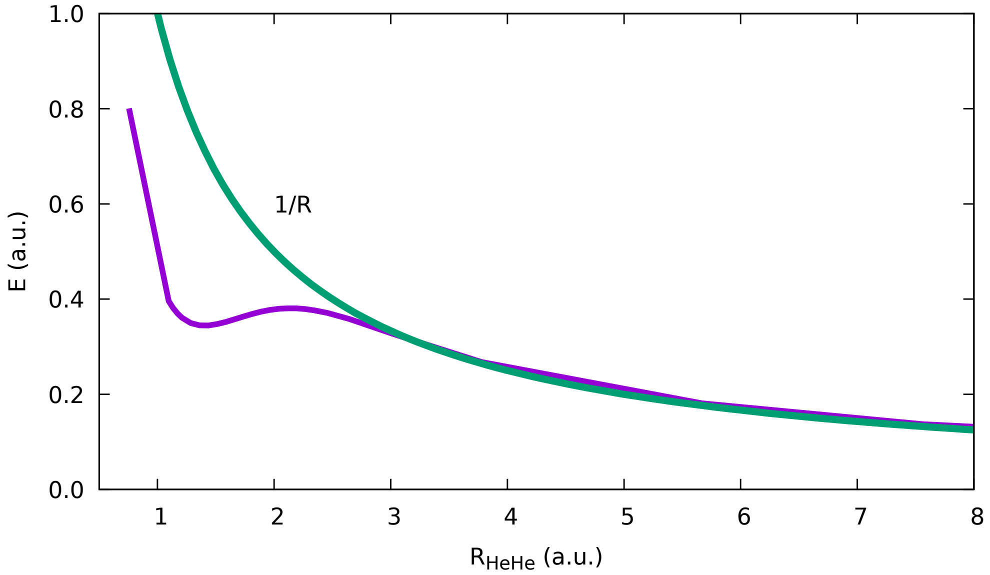

Supplementary Note 4. The He_2^{2+} ion

Supplementary Fig. 1 shows the energy of the He_2^{2+} ion as a function of the interatomic distance. Also the IQA atomic $T^A, E^A_{self}, E^{AB}_{int}, V^{AB}_{xc}, V^{AB}_{cl}, Q^A, \lambda^A, \delta^{AB}$ values, in a.u. are shown below.

#R/2	KinA	NetA	Interaction	XC	Classic	QA	LocA	DI
0.25	2.172862	-2.157279	0.808111	-0.414656	1.222767	0.999966	0.568182	0.863770
0.27	2.072610	-2.135915	0.704909	-0.389591	1.094500	0.999934	0.576925	0.846413
0.29	1.989737	-2.114550	0.622740	-0.365845	0.988585	1.000082	0.586388	0.826895
0.31	1.922188	-2.094106	0.556356	-0.343349	0.899705	1.000098	0.597008	0.805590
0.32	1.893566	-2.084402	0.527954	-0.332512	0.860466	0.999996	0.602852	0.794311
0.33	1.867774	-2.075134	0.502858	-0.321722	0.824580	1.000008	0.608883	0.782200
0.34	1.844714	-2.066445	0.480365	-0.311094	0.791459	1.000020	0.615231	0.769459
0.35	1.824376	-2.057694	0.461219	-0.300298	0.761517	1.000188	0.621750	0.755748
0.36	1.806820	-2.049660	0.443231	-0.289945	0.733176	1.000035	0.628929	0.742000
0.37	1.791146	-2.042343	0.427732	-0.279510	0.707242	1.000096	0.636243	0.727128
0.38	1.778164	-2.035258	0.413646	-0.269308	0.682954	0.999904	0.644166	0.712054
0.39	1.766920	-2.028717	0.401858	-0.258948	0.660806	0.999947	0.652212	0.695789
0.40	1.757675	-2.022737	0.391443	-0.248701	0.640144	0.999932	0.660676	0.678919
0.41	1.750272	-2.017023	0.382958	-0.238332	0.621290	1.000054	0.669362	0.661059
0.42	1.744843	-2.012094	0.374774	-0.228355	0.603129	0.999869	0.678708	0.643108
0.43	1.740770	-2.007378	0.368950	-0.218028	0.586978	1.000079	0.688001	0.623683
0.44	1.738700	-2.003026	0.363580	-0.207985	0.571565	1.000023	0.697887	0.604136
0.45	1.737901	-1.999588	0.358721	-0.198163	0.556884	0.999916	0.708131	0.584074
0.46	1.738547	-1.996195	0.355450	-0.188184	0.543634	1.000010	0.718456	0.563048
0.47	1.740552	-1.993381	0.352563	-0.178450	0.531013	0.999995	0.729143	0.541735
0.48	1.743873	-1.990994	0.350132	-0.168919	0.519051	0.999901	0.740126	0.520146
0.49	1.748118	-1.988925	0.348833	-0.159347	0.508180	1.000032	0.751060	0.497753
0.50	1.753553	-1.987167	0.347814	-0.150025	0.497839	1.000071	0.762218	0.475281
0.51	1.759904	-1.985938	0.346851	-0.141030	0.487881	1.000010	0.773560	0.452842
0.52	1.767252	-1.984873	0.346167	-0.132279	0.478446	0.999893	0.784988	0.430453
0.53	1.775069	-1.984088	0.346257	-0.123642	0.469899	0.999994	0.796172	0.407681
0.54	1.783577	-1.983588	0.346285	-0.115326	0.461611	1.000007	0.807370	0.385234
0.55	1.792607	-1.983294	0.346416	-0.107311	0.453727	1.000009	0.818445	0.363075
0.56	1.802008	-1.983206	0.346621	-0.099610	0.446231	1.000020	0.829324	0.341271
0.58	1.742189	-1.981339	0.346863	-0.084924	0.431787	1.000004	0.851309	0.297364

0.60	1.841447	-1.984245	0.347029	-0.072296	0.419325	1.000016	0.870057	0.259823
0.65	1.888208	-1.987121	0.344507	-0.046343	0.390850	0.999989	0.912073	0.175897
0.70	1.925897	-1.990108	0.337549	-0.028688	0.366237	0.999989	0.942818	0.114407
0.80	1.970757	-1.994224	0.314079	-0.010540	0.324619	1.000010	0.977079	0.045802
0.90	1.988738	-1.996176	0.286584	-0.003955	0.290539	1.000009	0.990754	0.018457
1.50	1.998666	-1.998006	0.175902	-0.000049	0.175951	1.000006	0.999835	0.000304
2.50	1.998865	-1.998124	0.105778	0.000000	0.105778	1.000006	0.999987	0.000000
3.00	1.998879	-1.998132	0.088169	0.000000	0.088169	1.000006	0.999987	0.000000
3.50	1.998900	-1.998121	0.075574	-0.000004	0.075578	0.999999	0.999987	0.000031
4.00	1.998889	-1.998136	0.066139	0.000000	0.066139	1.000006	0.999987	0.000000
5.00	1.998891	-1.998138	0.052915	0.000000	0.052915	1.000006	0.999987	0.000000
5.50	1.998892	-1.998138	0.048105	0.000000	0.048105	1.000006	0.999987	0.000000
6.00	1.998892	-1.998138	0.044097	0.000000	0.044097	1.000006	0.999987	0.000000
10.00	1.998893	-1.998139	0.026459	0.000000	0.026459	1.000006	0.999987	0.000000

Supplementary Note 5. The LiF molecule

IQA data for the S0 and S1 states (All data in a.u.)

S0

R	Te	Vee	Ven	Te	Vee	Ven	V^{Li.F}_{nn}	V^{Li.F}_{en}	V^{Li.F}_{ne}	V^{Li.F}_{ee}	V^{Li.F}_{int}
1.40	7.429712	1.812149	-16.449403	99.711313	44.142268	-243.467498	10.205556	-7.010065	-11.122650	7.580076	-0.347082
1.60	7.343949	1.761228	-16.329994	99.537086	43.955841	-243.130365	8.929862	-6.112139	-9.824324	6.684704	-0.321898
2.00	7.269939	1.706898	-16.215048	99.40554	43.726690	-242.781499	7.143889	-4.850854	-7.940246	5.373712	-0.273498
2.50	7.248059	1.685015	-16.174885	99.358549	43.571391	-242.583274	5.715112	-3.866514	-6.375749	4.304689	-0.222462
3.00	7.243971	1.684888	-16.172372	99.344823	43.479660	-242.483149	4.762593	-3.239452	-5.302084	3.598518	-0.180424
4.00	7.233762	1.66951	-16.145138	99.352607	43.488627	-242.497771	3.571945	-2.409949	-3.978484	2.680857	-0.135632
4.50	7.232778	1.668553	-16.143125	99.356475	43.505606	-242.519364	3.175062	-2.142949	-3.531663	2.380653	-0.118896
5.00	7.232466	1.668826	-16.143294	99.359562	43.519598	-242.536926	2.857556	-1.930941	-3.174209	2.142018	-0.105577
5.50	7.23296	1.671136	-16.1469	99.361213	43.515929	-242.534799	2.597778	-1.760024	-2.881840	1.949376	-0.094710
6.00	7.236249	1.681532	-16.163882	99.359803	43.447696	-242.463375	2.381296	-1.627224	-2.635840	1.796887	-0.084880
6.20	7.240756	1.695493	-16.186762	99.356476	43.348251	-242.358065	2.304480	-1.592059	-2.544723	1.752182	-0.080119
6.40	7.253688	1.735359	-16.252127	99.346417	43.061419	-242.053870	2.232465	-1.589859	-2.449039	1.734179	-0.072253
6.50	7.270331	1.78648	-16.335958	99.333557	42.694149	-241.664324	2.19812	-1.625222	-2.391201	1.753855	-0.064447
6.60	7.304287	1.890422	-16.506422	99.307796	41.950087	-240.875337	2.164815	-1.720090	-2.314657	1.819843	-0.050089
6.70	7.356372	2.049179	-16.766811	99.269316	40.819419	-239.676847	2.132504	-1.873655	-2.219504	1.931439	-0.029216
6.80	7.397525	2.173949	-16.971582	99.239905	39.936045	-238.741496	2.101144	-1.983710	-2.140117	2.009374	-0.013309
7.00	7.425051	2.256484	-17.106919	99.221148	39.357049	-238.127927	2.041111	-2.015157	-2.048849	2.020192	-0.002703
7.20	7.430614	2.272859	-17.133810	99.217726	39.244126	-238.008540	1.984414	-1.975536	-1.986243	1.976690	-0.000676
8.00	7.433153	2.280146	-17.145805	99.216447	39.195150	-237.956942	1.785972	-1.784114	-1.785471	1.783840	0.000227
10.00	7.433267	2.280483	-17.146365	99.216563	39.193028	-237.954870	1.428778	-1.427325	-1.428458	1.427243	0.000238

S1

R	Te	Vee	Ven	Te	Vee	Ven	V^{Li.F}_{nn}	V^{Li.F}_{en}	V^{Li.F}_{ne}	V^{Li.F}_{ee}	V^{Li.F}_{int}
1.40	7.511937	2.302677	-17.153165	99.404666	39.598151	-238.537147	10.205556	-8.476269	-10.098822	8.341335	-0.028200
1.60	7.425630	2.245921	-17.059404	99.332961	39.555757	-238.407302	8.929862	-7.458130	-8.923702	7.425689	-0.026281
1.80	7.410039	2.234247	-17.042071	99.281884	39.486805	-238.295698	7.937655	-6.734352	-7.954136	6.728842	-0.021992
2.00	7.418309	2.239102	-17.052896	99.249622	39.432787	-238.223525	7.143889	-6.176104	-7.156412	6.170755	-0.017872
2.50	7.416076	2.230188	-17.055313	99.251955	39.475161	-238.264472	5.715112	-5.078300	-5.746147	5.093991	-0.015345
3.00	7.421520	2.229908	-17.064450	99.256390	39.545840	-238.340365	4.762593	-4.325407	-4.803703	4.350335	-0.016182
3.50	7.430711	2.247457	-17.093205	99.246502	39.501385	-238.290936	4.082223	-3.840595	-4.104712	3.852659	-0.010425
4.00	7.431148	2.252959	-17.102746	99.239027	39.464479	-238.250438	3.571945	-3.425801	-3.589491	3.435427	-0.007920
4.50	7.429293	2.255978	-17.107585	99.233509	39.427250	-238.208361	3.175062	-3.079024	-3.189762	3.087430	-0.006294
5.00	7.428776	2.258677	-17.111437	99.228590	39.391879	-238.168976	2.857556	-2.795018	-2.868698	2.801407	-0.004753
5.50	7.427961	2.258349	-17.110381	99.225883	39.381779	-238.156653	2.597778	-2.551985	-2.607379	2.557746	-0.003840
6.00	7.424140	2.248489	-17.094075	99.227267	39.445235	-238.223506	2.381296	-2.331292	-2.394169	2.339536	-0.004629
6.20	7.419504	2.234627	-17.071321	99.230647	39.543860	-238.328106	2.304480	-2.239959	-2.322625	2.251761	-0.006343
6.40	7.406466	2.194845	-17.006076	99.240764	39.830102	-238.631820	2.232465	-2.123244	-2.265858	2.145238	-0.011399
6.50	7.389755	2.143817	-16.922386	99.253700	40.196961	-239.021009	2.198120	-2.031344	-2.250945	2.066327	-0.017842
6.60	7.355674	2.040112	-16.752276	99.279565	40.939740	-239.808761	2.164815	-1.882391	-2.256708	1.943480	-0.030804
6.70	7.303407	1.881761	-16.492492	99.318225	42.068104	-241.004978	2.132504	-1.677447	-2.282944	1.777629	-0.050258
6.80	7.262090	1.757159	-16.288047	99.347826	42.950423	-241.939926	2.101144	-1.516419	-2.295810	1.646146	-0.064939
7.00	7.234595	1.674713	-16.152748	99.366553	43.528448	-242.551991	2.041111	-1.386060	-2.260052	1.532011	-0.072989
7.20	7.229053	1.658217	-16.125675	99.369995	43.642027	-242.672122	1.984414	-1.330798	-2.203040	1.476547	-0.072878
8.00	7.226526	1.650754	-16.113427	99.371355	43.692199	-242.725128	1.785972	-1.190979	-1.984898	1.323595	-0.066309
10.00	7.226394	1.650377	-16.112808	99.371531	43.695146	-242.728384	1.428778	-0.952518	-1.587761	1.058505	-0.052995

Supplementary Note 6. The dissociation of acetylene

IQA

CASSCF/def2-TVZPP IQA data (all but distances in a.u.)

#dCC [Angs]	E_selfC	E_selfH	E_intCC	E_CC^cl	E_CC^xc	E_intCH	E_CH^cl	E_CH^xc	E_intCH'	E_CH'^cl	E_CH'^xc	E_intHH	E_HH^cl	E_HH^xc	Etotal
1.1999	-37.619072	-0.447876	-0.435978	+0.130265	-0.566243	-0.209392	+0.042563	-0.251954	-0.007337	+0.002072	-0.009409	+0.002354	+0.003874	-0.001520	-77.000978
1.2155	-37.621257	-0.447247	-0.434025	+0.122496	-0.556521	-0.208918	+0.042752	-0.251670	-0.007593	+0.001752	-0.009344	+0.002479	+0.003987	-0.001509	-77.001574
1.2499	-37.625489	-0.445895	-0.427069	+0.107358	-0.534427	-0.208185	+0.043047	-0.251233	-0.008084	+0.001114	-0.009198	+0.002736	+0.004221	-0.001485	-76.999639
1.2999	-37.629639	-0.443982	-0.415007	+0.088898	-0.503905	-0.207156	+0.043354	-0.250510	-0.008783	+0.000196	-0.008979	+0.003090	+0.004543	-0.001453	-76.991036
1.3999	-37.633845	-0.442588	-0.379021	+0.058407	-0.437428	-0.204855	+0.044379	-0.249234	-0.009942	-0.001108	-0.008834	+0.003044	+0.004479	-0.001435	-76.958436
1.4499	-37.634772	-0.443581	-0.358238	+0.045661	-0.403899	-0.203679	+0.045109	-0.248788	-0.010206	-0.001240	-0.008966	+0.002580	+0.004007	-0.001427	-76.940134
1.4999	-37.634936	-0.444389	-0.338004	+0.036402	-0.374406	-0.203022	+0.045538	-0.248560	-0.010310	-0.001294	-0.009016	+0.002220	+0.003618	-0.001398	-76.921098
1.5499	-37.634654	-0.445092	-0.318918	+0.029153	-0.348072	-0.202678	+0.045780	-0.248458	-0.010360	-0.001363	-0.008997	+0.001925	+0.003286	-0.001362	-76.902562
1.5999	-37.634014	-0.445654	-0.300289	+0.023516	-0.323805	-0.202447	+0.045997	-0.248444	-0.010294	-0.001388	-0.008906	+0.001688	+0.003009	-0.001321	-76.883417
1.6499	-37.633170	-0.446133	-0.282619	+0.018939	-0.301558	-0.202298	+0.046153	-0.248452	-0.010200	-0.001436	-0.008765	+0.001494	+0.002774	-0.001279	-76.864727
1.6999	-37.632216	-0.446507	-0.265615	+0.015275	-0.280889	-0.202242	+0.046283	-0.248526	-0.010063	-0.001477	-0.008586	+0.001335	+0.002573	-0.001238	-76.846336
1.7499	-37.631418	-0.446781	-0.249356	+0.012269	-0.261625	-0.202321	+0.046299	-0.248620	-0.009880	-0.001506	-0.008374	+0.001205	+0.002402	-0.001197	-76.828951
1.7999	-37.630528	-0.447018	-0.233910	+0.009854	-0.243764	-0.202437	+0.046376	-0.248812	-0.009688	-0.001549	-0.008139	+0.001092	+0.002249	-0.001157	-76.812158
1.8499	-37.629750	-0.447194	-0.219198	+0.007900	-0.227097	-0.202650	+0.046387	-0.249037	-0.009478	-0.001587	-0.007891	+0.000996	+0.002113	-0.001117	-76.796343
1.8999	-37.629143	-0.447330	-0.205009	+0.006338	-0.211347	-0.202835	+0.046420	-0.249255	-0.009237	-0.001608	-0.007628	+0.000917	+0.001994	-0.001076	-76.781183
1.9499	-37.628697	-0.447419	-0.191563	+0.005069	-0.196632	-0.203088	+0.046409	-0.249497	-0.008989	-0.001632	-0.007356	+0.000849	+0.001885	-0.001036	-76.767100
1.9999	-37.628200	-0.447521	-0.178765	+0.004093	-0.182859	-0.203327	+0.046422	-0.249749	-0.008728	-0.001652	-0.007076	+0.000789	+0.001783	-0.000993	-76.753526
2.0499	-37.627936	-0.447597	-0.166500	+0.003307	-0.169807	-0.203701	+0.046379	-0.250080	-0.008442	-0.001657	-0.006785	+0.000737	+0.001687	-0.000950	-76.741118
2.0999	-37.627810	-0.447691	-0.154843	+0.002661	-0.157504	-0.203972	+0.046381	-0.250353	-0.008136	-0.001649	-0.006487	+0.000689	+0.001593	-0.000904	-76.729372
2.1499	-37.627826	-0.447821	-0.143610	+0.002141	-0.145751	-0.204343	+0.046336	-0.250679	-0.007806	-0.001626	-0.006180	+0.000646	+0.001503	-0.000857	-76.718556
2.1999	-37.627878	-0.447988	-0.132970	+0.001727	-0.134697	-0.204788	+0.046256	-0.251044	-0.007460	-0.001594	-0.005866	+0.000602	+0.001409	-0.000807	-76.708596
2.2499	-37.627984	-0.448200	-0.122799	+0.001370	-0.124169	-0.205268	+0.046158	-0.251426	-0.007092	-0.001546	-0.005546	+0.000558	+0.001312	-0.000754	-76.699329
2.2999	-37.628151	-0.448490	-0.113077	+0.001058	-0.114134	-0.205859	+0.045977	-0.251836	-0.006699	-0.001480	-0.005219	+0.000511	+0.001211	-0.000700	-76.690964
2.3499	-37.628422	-0.448872	-0.103743	+0.000708	-0.104452	-0.206257	+0.045869	-0.252126	-0.006269	-0.001385	-0.004885	+0.000457	+0.001101	-0.000644	-76.682927
2.3999	-37.628780	-0.449402	-0.094721	+0.000366	-0.095087	-0.207022	+0.045581	-0.252603	-0.005816	-0.001272	-0.004544	+0.000394	+0.000980	-0.000586	-76.676368
2.4499	-37.628955	-0.450098	-0.086037	-0.000053	-0.085983	-0.207703	+0.045250	-0.252953	-0.005329	-0.001130	-0.004199	+0.000317	+0.000844	-0.000528	-76.669891
2.4999	-37.629166	-0.451052	-0.077449	-0.000576	-0.076873	-0.208653	+0.044711	-0.253364	-0.004787	-0.000947	-0.003840	+0.000220	+0.000691	-0.000471	-76.664544
2.5499	-37.629218	-0.452367	-0.068878	-0.001304	-0.067574	-0.209564	+0.043972	-0.253536	-0.004169	-0.000710	-0.003459	+0.000102	+0.000518	-0.000416	-76.659410
2.5999	-37.629136	-0.454407	-0.059406	-0.002359	-0.057047	-0.211049	+0.042594	-0.253643	-0.003399	-0.000393	-0.003006	-0.000055	+0.000313	-0.000368	-76.655441
2.6499	-37.629255	-0.457581	-0.046284	-0.003986	-0.042298	-0.214012	+0.040016	-0.254027	-0.002172	+0.000075	-0.002247	-0.000211	+0.000101	-0.000312	-76.652534
2.6999	-37.630435	-0.458400	-0.038998	-0.004115	-0.034883	-0.215421	+0.039188	-0.254609	-0.001607	+0.000186	-0.001793	-0.000197	+0.000060	-0.000257	-76.650921
2.7499	-37.631322	-0.458772	-0.033919	-0.004025	-0.029894	-0.216299	+0.038790	-0.255090	-0.001253	+0.000230	-0.001483	-0.000174	+0.000042	-0.000216	-76.649385
2.7999	-37.632268	-0.458997	-0.029823	-0.003881	-0.025942	-0.217045	+0.038478	-0.255524	-0.000989	+0.000254	-0.001243	-0.000153	+0.000031	-0.000183	-76.648573
2.8499	-37.632994	-0.459155	-0.026426	-0.003726	-0.022700	-0.217597	+0.038239	-0.255836	-0.000783	+0.000266	-0.001049	-0.000134	+0.000022	-0.000157	-76.647620
2.8999	-37.633633	-0.459319	-0.023605	-0.003573	-0.020033	-0.218030	+0.038033	-0.256063	-0.000618	+0.000268	-0.000886	-0.000119	+0.000017	-0.000136	-76.646926
2.9499	-37.634339	-0.459420	-0.021047	-0.003406	-0.017641	-0.218464	+0.037868	-0.256332	-0.000486	+0.000271	-0.000757	-0.000104	+0.000012	-0.000116	-76.646570
2.9999	-37.634822	-0.459501	-0.018935	-0.003268	-0.015667	-0.218788	+0.037733	-0.256521	-0.000378	+0.000267	-0.000645	-0.000092	+0.000009	-0.000101	-76.646006
3.3499	-37.637312	-0.459847	-0.009536	-0.002366	-0.007170	-0.220083	+0.037089	-0.257172	-0.000003	+0.000220	-0.000223	-0.000039	+0.000002	-0.000040	-76.644065
3.7499	-37.638491	-0.460024	-0.004848	-0.001649	-0.003199	-0.220611	+0.036756	-0.257367	+0.000104	+0.000168	-0.000064	-0.000017	+0.000001	-0.000019	-76.642908
4.1999	-37.639209	-0.460089	-0.002410	-0.001128	-0.001282	-0.221008	+0.036509	-0.257518	+0.000132	+0.000150	-0.000018	-0.000004	+0.000002	-0.000005	-76.642763
4.5999	-37.639310	-0.460132	-0.001428	-0.000819	-0.000609	-0.221080	+0.036404	-0.257485	+0.000109	+0.000110	-0.000001	-0.000002	+0.000003	-0.000005	-76.642255
4.9999	-37.639393	-0.460186	-0.000900	-0.000617	-0.000283	-0.221133	+0.036316	-0.257449	+0.000091	+0.000088	+0.000004	+0.000002	+0.000004	-0.000003	-76.642140

EDFs

Electron distribution functions. Distance in Å.
A=CH, B=CH

# D(C-C)	p(10,4)	p(9,5)	p(8,6)	p(7,7)	p(6,8)	p(5,9)	p(4,10)
0.5499	0.0102354	0.0793986	0.2395801	0.3411076	0.2396127	0.0794279	0.0102458
1.1999	0.0036916	0.0549434	0.2437186	0.3954753	0.2435791	0.0548870	0.0036880
1.2499	0.0032756	0.0522539	0.2433955	0.4022265	0.2433366	0.0522261	0.0032723
1.2999	0.0028832	0.0494689	0.2428587	0.4094798	0.2429230	0.0494918	0.0028841
1.3499	0.0025174	0.0466261	0.2421721	0.4173150	0.2422031	0.0466393	0.0025189
1.3999	0.0020130	0.0424274	0.2413678	0.4282789	0.2414348	0.0424544	0.0020157
1.4499	0.0016131	0.0384790	0.2402025	0.4394617	0.2401690	0.0384572	0.0016096
1.4999	0.0013048	0.0349241	0.2386475	0.4504482	0.2384956	0.0348723	0.0013006
1.5499	0.0010557	0.0315934	0.2365808	0.4615243	0.2365876	0.0315961	0.0010563
1.5999	0.0008571	0.0285346	0.2342411	0.4727640	0.2342084	0.0285319	0.0008580
1.6499	0.0006975	0.0256949	0.2314945	0.4842406	0.2314841	0.0256876	0.0006968
1.6999	0.0005661	0.0230468	0.2283544	0.4960508	0.2283698	0.0230440	0.0005650
1.7499	0.0004570	0.0205895	0.2248419	0.5082551	0.2248155	0.0205817	0.0004566
1.7999	0.0003687	0.0183228	0.2208763	0.5208389	0.2208982	0.0183246	0.0003685
1.8499	0.0002952	0.0162190	0.2164259	0.5339030	0.2166000	0.0162585	0.0002968
1.8999	0.0002376	0.0143467	0.2117088	0.5473856	0.2117292	0.0143529	0.0002379
1.9499	0.0001904	0.0126333	0.2064884	0.5613943	0.2064720	0.0126303	0.0001904
1.9999	0.0001522	0.0110783	0.2007714	0.5759403	0.2008227	0.0110822	0.0001522
2.0499	0.0001200	0.0096534	0.1945818	0.5911313	0.1947061	0.0096857	0.0001212
2.0999	0.0000965	0.0084366	0.1880862	0.6067734	0.1880806	0.0084301	0.0000962
2.1499	0.0000768	0.0073354	0.1811575	0.6230721	0.1809745	0.0073072	0.0000761
2.1999	0.0000607	0.0063392	0.1736726	0.6400190	0.1735298	0.0063185	0.0000602
2.2499	0.0000477	0.0054520	0.1657312	0.6576120	0.1656669	0.0054427	0.0000474
2.2999	0.0000366	0.0046394	0.1572316	0.6759432	0.1574385	0.0046733	0.0000373
2.3499	0.0000299	0.0040131	0.1487198	0.6946085	0.1486138	0.0039856	0.0000292

2.3999	0.0000228	0.0033876	0.1394350	0.7143200	0.1394277	0.0033842	0.0000228
2.4499	0.0000178	0.0028608	0.1297036	0.7348007	0.1297428	0.0028565	0.0000177
2.4999	0.0000133	0.0023678	0.1191955	0.7567017	0.1193220	0.0023859	0.0000136
2.5499	0.0000105	0.0019684	0.1078405	0.7804168	0.1077915	0.0019619	0.0000104
2.5999	0.0000077	0.0015591	0.0937494	0.8094577	0.0936658	0.0015526	0.0000077
2.6499	0.0000048	0.0010512	0.0712126	0.8553522	0.0713144	0.0010599	0.0000049
2.6999	0.0000033	0.0007933	0.0596060	0.8792700	0.0595330	0.0007912	0.0000033
2.7499	0.0000024	0.0006212	0.0516298	0.8955596	0.0515670	0.0006177	0.0000024
2.7999	0.0000017	0.0004906	0.0452705	0.9085124	0.0452337	0.0004893	0.0000017
2.8499	0.0000013	0.0003922	0.0400214	0.9191952	0.0399969	0.0003917	0.0000013
2.8999	0.0000009	0.0003181	0.0356735	0.9280705	0.0356198	0.0003163	0.0000009
2.9499	0.0000007	0.0002546	0.0317208	0.9360584	0.0317098	0.0002551	0.0000007
2.9999	0.0000005	0.0002068	0.0284094	0.9427571	0.0284180	0.0002077	0.0000005
3.3499	0.0000001	0.0000526	0.0138696	0.9721499	0.0138749	0.0000528	0.0000001
3.7499	0.0000000	0.0000131	0.0067114	0.9866561	0.0066069	0.0000125	0.0000000
4.1999	0.0000000	0.0000019	0.0026811	0.9944561	0.0028585	0.0000024	0.0000000
4.5999	0.0000000	0.0000004	0.0012922	0.9972923	0.0014145	0.0000006	0.0000000
4.9999	0.0000000	0.0000001	0.0006218	0.9987054	0.0006726	0.0000001	0.0000000

Spin Partition for $n_{\mathrm{CH}} = 7$. (Aα Aβ, Bα Bβ)

# D(CC)	(4,3,3,4)	(3,4,4,3)	(5,2,2,5)	(2,5,5,2)	total
0.5499	0.4412753	0.4412753	0.0587185	0.0587185	0.3411076
0.9499	0.4363310	0.4363310	0.0636687	0.0636687	0.1990421
0.9999	0.4349134	0.4349134	0.0650863	0.0650863	0.2242285
1.0499	0.4332028	0.4332028	0.0667970	0.0667970	0.2555467
1.0999	0.4311243	0.4311243	0.0688755	0.0688755	0.2942772
1.1499	0.4283689	0.4283689	0.0716310	0.0716310	0.3600347
1.1999	0.4254491	0.4254491	0.0745508	0.0745508	0.3954753
1.2499	0.4225222	0.4225222	0.0774777	0.0774777	0.4022265
1.2999	0.4191995	0.4191995	0.0808004	0.0808004	0.4094798
1.3499	0.4154364	0.4154364	0.0845636	0.0845636	0.4173150
1.3999	0.4136878	0.4136878	0.0863119	0.0863119	0.4282789
1.4499	0.4115535	0.4115535	0.0884460	0.0884460	0.4394617
1.4999	0.4088901	0.4088901	0.0911093	0.0911093	0.4504482
1.5499	0.4060641	0.4060641	0.0939351	0.0939351	0.4615243
1.5999	0.4031566	0.4031566	0.0968426	0.0968426	0.4727640
1.6499	0.4003015	0.4003015	0.0996976	0.0996976	0.4842406
1.6999	0.3975410	0.3975410	0.1024580	0.1024580	0.4960508
1.7499	0.3949041	0.3949041	0.1050950	0.1050950	0.5082551
1.7999	0.3924419	0.3924419	0.1075573	0.1075573	0.5208389
1.8499	0.3901923	0.3901923	0.1098069	0.1098069	0.5339030
1.8999	0.3882043	0.3882043	0.1117950	0.1117950	0.5473856
1.9499	0.3864953	0.3864953	0.1135040	0.1135040	0.5613943
1.9999	0.3851300	0.3851300	0.1148694	0.1148694	0.5759403
2.0499	0.3841536	0.3841536	0.1158459	0.1158459	0.5911313
2.0999	0.3836504	0.3836504	0.1163491	0.1163491	0.6067734
2.1499	0.3837053	0.3837053	0.1162943	0.1162943	0.6230721
2.1999	0.3844220	0.3844220	0.1155776	0.1155776	0.6400190
2.2499	0.3859477	0.3859477	0.1140519	0.1140519	0.6576120
2.2999	0.3884769	0.3884769	0.1115228	0.1115228	0.6759432
2.3499	0.3922974	0.3922974	0.1077023	0.1077023	0.6946085
2.3999	0.3977603	0.3977603	0.1022395	0.1022395	0.7143200
2.4499	0.4053915	0.4053915	0.0946083	0.0946083	0.7348007
2.4999	0.4159910	0.4159910	0.0840089	0.0840089	0.7567017
2.5499	0.4308753	0.4308753	0.0691246	0.0691246	0.7804168
2.5999	0.4536336	0.4536336	0.0463664	0.0463664	0.8094577
2.6499	0.4877717	0.4877717	0.0122283	0.0122283	0.8553522
2.6999	0.4943656	0.4943656	0.0056344	0.0056344	0.8792700
2.7499	0.4966824	0.4966824	0.0033175	0.0033175	0.8955596
2.7999	0.4978744	0.4978744	0.0021256	0.0021256	0.9085124

2.8499	0.4985756	0.4985756	0.0014244	0.0014244	0.9191952
2.8999	0.4990096	0.4990096	0.0009904	0.0009904	0.9280705
2.9499	0.4993099	0.4993099	0.0006901	0.0006901	0.9360584
2.9999	0.4995058	0.4995058	0.0004942	0.0004942	0.9427571
3.3499	0.4999417	0.4999417	0.0000583	0.0000583	0.9721499
3.7499	0.4999928	0.4999928	0.0000072	0.0000072	0.9866561
4.1999	0.4999992	0.4999992	0.0000008	0.0000008	0.9944561
4.5999	0.4999998	0.4999998	0.0000002	0.0000002	0.9972923
4.9999	0.5000000	0.5000000	0.0000000	0.0000000	0.9987054

Supplementary Note 7. $LiBe^+$

summary of the HF/6-311G(p) IQA and EDF data follows. All data in a.u. Li=atom 1, Be=atom 2.

```
Atomic Contributions for neq:  1
        Atom number          :  1
 --------------------------------------------------------------------------------
 kinetic energy          =      7.27702800
 potential energy        =    -14.54668304
 electron repulsion      =      1.72072578
 ---coulomb              =               3.37518698
 ---exch+corr            =              -1.65446120
 ---self                 =               1.64948462
 el-own-nuc attraction   =    -16.24330864
 net energy              =     -7.24555486
 interaction energy      =     -0.04820036
 additive energy         =     -7.26965504
 effective energy        =     -7.29375522
 2T+V                    =      0.00737296
 Int rho_2               =      2.22102822
 Integ rho_2 J           =      4.21815128
 Integ rho_2 XC   (F_AA) =      1.99712307
 SUM-RULE-TEST           =      2.05379695  AND SHOULD BE      2.05381384

 Additive Position Spread Tensor
       0.402614
       0.000000       0.402614
      -0.000000      -0.000000       0.761932


 ===================  Interaction with atom:   2  =================================================
 (NN,EN,NE,EE,Inter)            2.40012114    -1.64660652    -2.50729075     1.70557576    -0.04820036
 EE  wself : (coul,XC,self)     1.72024747    -0.01467171     0.01469112
 EE  woself: (coul,XC)          1.70555635     0.00001942
 Coul comp.: (longr, shortr)   0.170562E+01  0.146234E-01
 Classical Int. (Long,Total)  -0.740127E-01 -0.335287E-01
 RHO_2 Integ comp (TOT,J,XC)      8.04783426     8.10450814     0.05667388
 F_AB (XC)                      0.11334776

Atomic Contributions for neq:  2
        Atom number          :  2
 --------------------------------------------------------------------------------
 kinetic energy          =     14.55176466
 potential energy        =    -29.11205317
 electron repulsion      =      4.44977950
 ---coulomb              =               7.11137255
 ---exch+corr            =              -2.66159304
 ---self                 =               2.61255737
 el-own-nuc attraction   =    -33.53773249
 net energy              =    -14.53618834
 interaction energy      =     -0.04820036
 additive energy         =    -14.56028852
 effective energy        =    -14.58438870
 2T+V                    =     -0.00852386
 Int rho_2               =     11.68221453
 Integ rho_2 J           =     15.57152597
 Integ rho_2 XC   (F_AA) =      3.88931143
 SUM-RULE-TEST           =      3.94598531  AND SHOULD BE      3.94607729
```

```
--------------------------------------------------------------------------------
 Atom      Charge      Eadd         Enet         Eeff          Kin     Loc Index
 --------------------------------------------------------------------------------
   1     0.946186    -7.269655    -7.245555    -7.293755     7.277028   1.997123
   2     0.053923   -14.560289   -14.536188   -14.584389    14.551765   3.889311

# M-BASINS ELECTRON NUMBER PROBABILITY DISTRIBUTION NOT INCLUDING SPIN
 #--------------------------------------------------------------------------
 # NUMBER OF GROUPS               =        2
 # TOTAL NUMBER OF PROBABILITIES  =        7
 #--------------------------------------------------------------------------
 #     Probability            n1    n2    n3 ...
 #     0.9426745280592217      2     4
 #     0.0543463145215340      3     3
 #     0.0020858010821647      1     5
 #     0.0007833328433824      4     2
 #     0.0000011538562734      0     6
 #     0.0000000000104736      5     1
 #     0.0000000000000000      6     0
 #--------------------------------------------------------------------------
 #    0.9998911303730498  <-- SUM,     7 PROBABILITIES > 0.0000000000E+00
```

Supplementary Note 8. The BeO case

Supplementary Fig. 2 shows that the BeO interaction starts with an O atom displaying a kind of σ-hole, clearly visible in the Laplacian of the density, around the bonding axis, which allows for a Be to O donor-acceptor σ favorable approach. The behavior of E_{self}^{Be} however evidence that as R decreases, we assist at a two step ionization process. In the first one, similarly to what was found in LiBe^{+}, a Be electron is transferred to the oxygen. This is half completed at about $R = 2.6$ Å, and at around 2.4 Å the second ionization of Be starts. These two stepwise ionizations are transparently unveiled in the evolution of E_{self}^{Be}. Supplementary Fig. 2 shows that E_{self}^{Be} depends linearly with the atomic electron population, with slopes equal to the Be first and second ionization energies. This is exactly what it is expect from grand canonical density functional theory.[16] Simultaneously, already at 2.6 Å two back-bonding π bonds involving the oxygen's electrons start to be clearly visible (natural adaptive orbitals are shown in Supplementary Fig. 5). Supplementary Fig. 3 unveils, with the help of UHF calculations, that the first ionization is a clear 2c,1e process, in which the two Be 2s electrons occupy different regions of space (in an *sp*-like hybridization geometry) and it is only the one pointing towards the internuclear region that delocalizes. This is in perfect consonance with the probabilities of the multiconfigurational probability distribution. Although it is not shown, the second ionization involves a similar process. An equivalent image to that offered by UHF is obtained by using rigorous one-electron functions obtained from spatial partitions (e.g. domain-averaged Fermi holes[17] or open-systems natural orbitals,[18], but this is out of the scope of this paper). Further analysis of the electron distribution shows that the contribution of triplet oxygen atoms is not negligible over a relatively small range of distances around $R \approx 1.9$ Å. Thanks to back-donation, at equilibrium, $Q(\mathrm{Be}) = 1.416$ e, although the Laplacian, for instance, shows neat quasi-spherical atomic shells, and $p(2, 10)$ is greater than 50%. Another very revealing point is that although during most of the association process the contribution of the σ link to the total bond order is considerably greater than that of the π ones, all the three contributions become basically equivalent after the second ionization is complete, pointing toward a rather isotropic final interaction. Summarizing, the Be to O σ donation soon evolves to include two π back-donating bonds, all this coupled to the Be to O ionization. After the second ionization starts, and surely at equilibrium, as evidenced by the self-energy analysis that clearly selects an in-the-molecule ionic reference, a better description of the system is that of an oxide anion donating rather symmetrically its σ and π electrons to a Be dication that acts as acceptor. In this regime, a dative, donor-acceptor bonding model is also admissible, but now the donor agent is the oxide anion, see the figure. Notice that as the Be-O distance decreases from the dissociation limit, a wealth of avoided crossings will occur that would allow a much more detailed account that the one here presented.

Evolution of the Laplacian

The behavior of $\nabla^2\rho$ at different interatomic distances is able to unveil subtle changes in the electron distribution. Supplementary Fig. 4 shows contour maps of the Laplacian on planes containing the nuclei.

Several interesting points can be highlighted. First, a charge concentration develops in the Be atom on the internuclear axis facing the O atom, clearly visible at $R = 5.0$ a.u., that disappears at $R = 3.8$, coinciding with the closing of the σ-hole along the internuclear axis in the O atom, a phenomenon ocurring between $R = 4.6$ and $R = 3.8$ a.u. Also clear is the development of a π-like torus around the Be atom after the first ionization has taken place, which is clearly related to the back-donation commented in the main text.

Evolution of the Natural Adaptive Orbitals

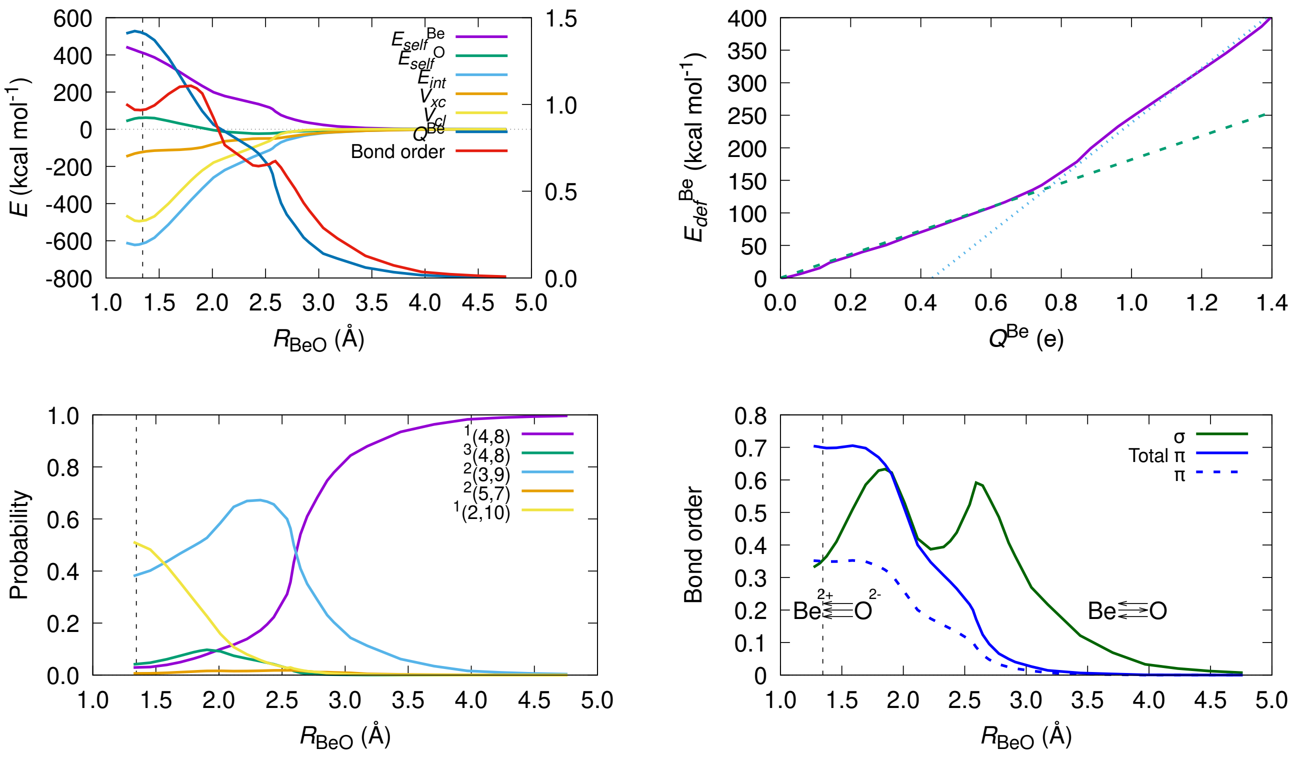

Supplementary Figure 2: CASSCF[8,8]/6-311G(p) spatial partitioning along the association process of a ^{1}S Be and a ^{1}D O atoms to form BeO. Top left: Spatial energy components. All energies referred to the infinite distance limit. Atomic charges and bond orders should be read on the right vertical axis, in au. Top right: E_{def} of the Be atom versus its atomic charge. The two straight lines have slopes equal to the first and second ionization energies of the Be atom. Bottom left: Probabilities of the different spin-resolved electron distributions. The spin multiplicity $2S + 1$ of any of the atoms in the distribution is indicated as a left superindex. Bottom right: Contribution of the σ and π channels to the total bond order. The two π equivalent channels have also added for convenience. The equilibrium geometry has been highlighted by a dashed vertical line.

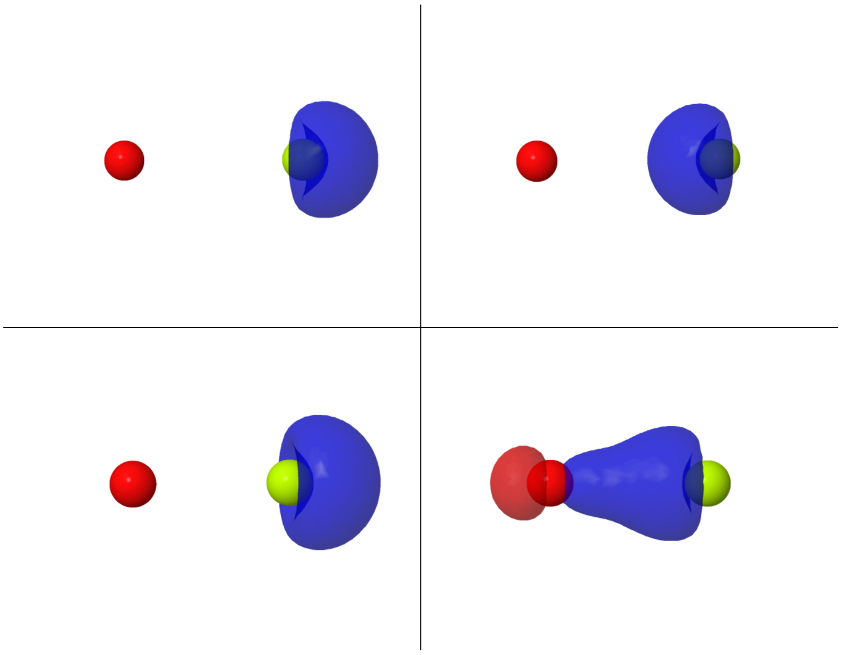

Supplementary Figure 3: UHF/6-311G(p) HOMOs in BeO for each spin channel, computed at $R_{\text{BeO}} = 3.2$ Å (top row) and $R_{\text{BeO}} = 2.4$ Å (bottom row), respectively. The Be atom is on the right, and $|\phi| = 0.1$ au.

Supplementary Fig. 5 shows the σ and π (only one of the two equivalent contributions) two-center natural adaptive orbitals of BeO at several distances.

The formation of the σ-bond as well as the π back-donation are clearly visible. Notice that at the equilibrium distance both the σ and π links are almost equally intense, showing that the separation is artificial and that there is almost spherical symmetry.

Supplementary Figure 4: BeO CAS[8,8]/6-311G(p) $\nabla^2\rho$ isocontours in a plane containing the Be and O nuclei at different R_{BeO} distances, in a.u. Negative isocontours are depicted in red, while positive in blue.

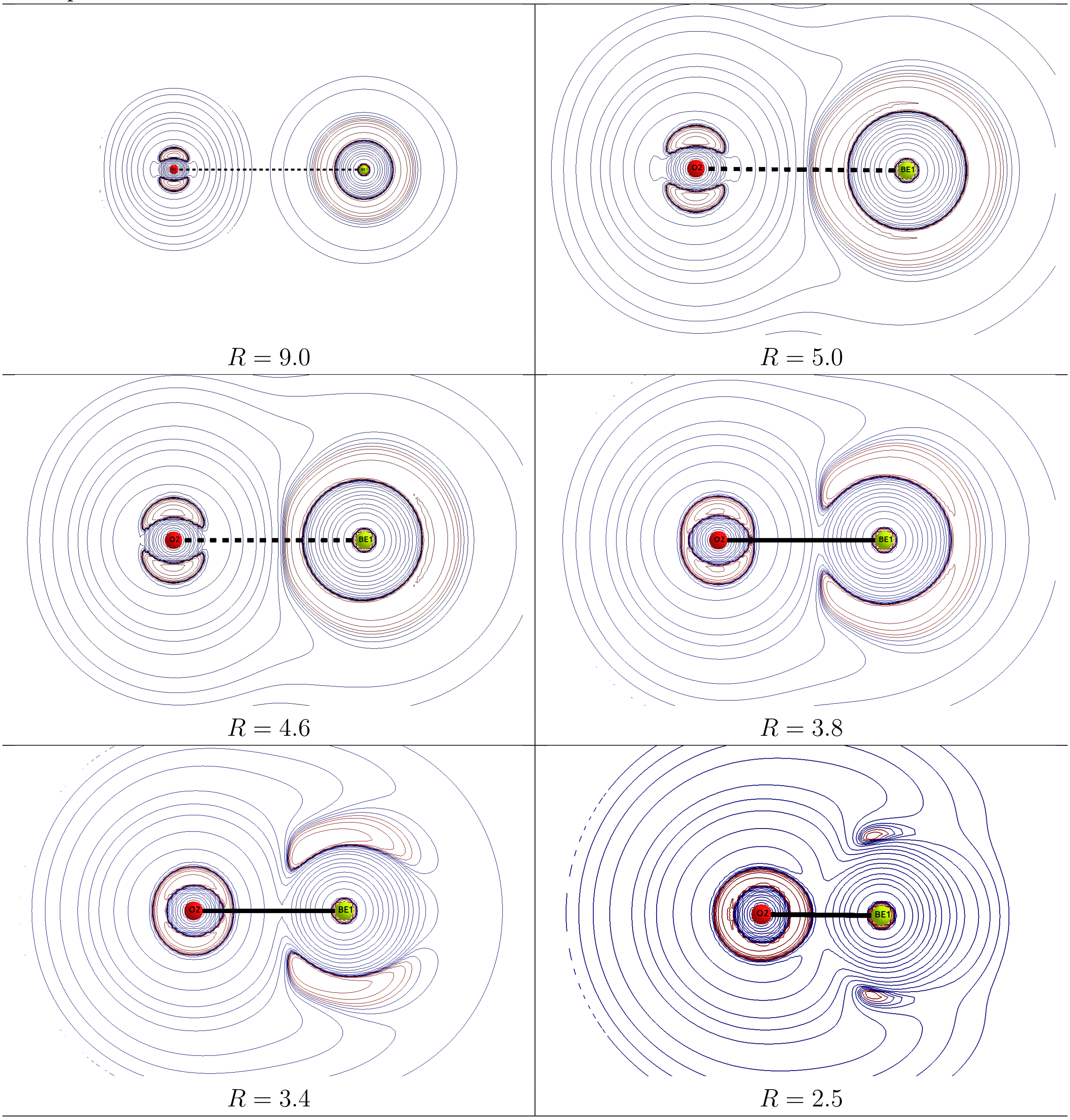

Supplementary Figure 5: BeO CAS[8,8]/6-311G(p) σ and π natural adaptive orbitals at several internuclear distances (in a.u.). The occupation numbers are shown in red. The contribution of each NAdO to the DI is twice this occupation.

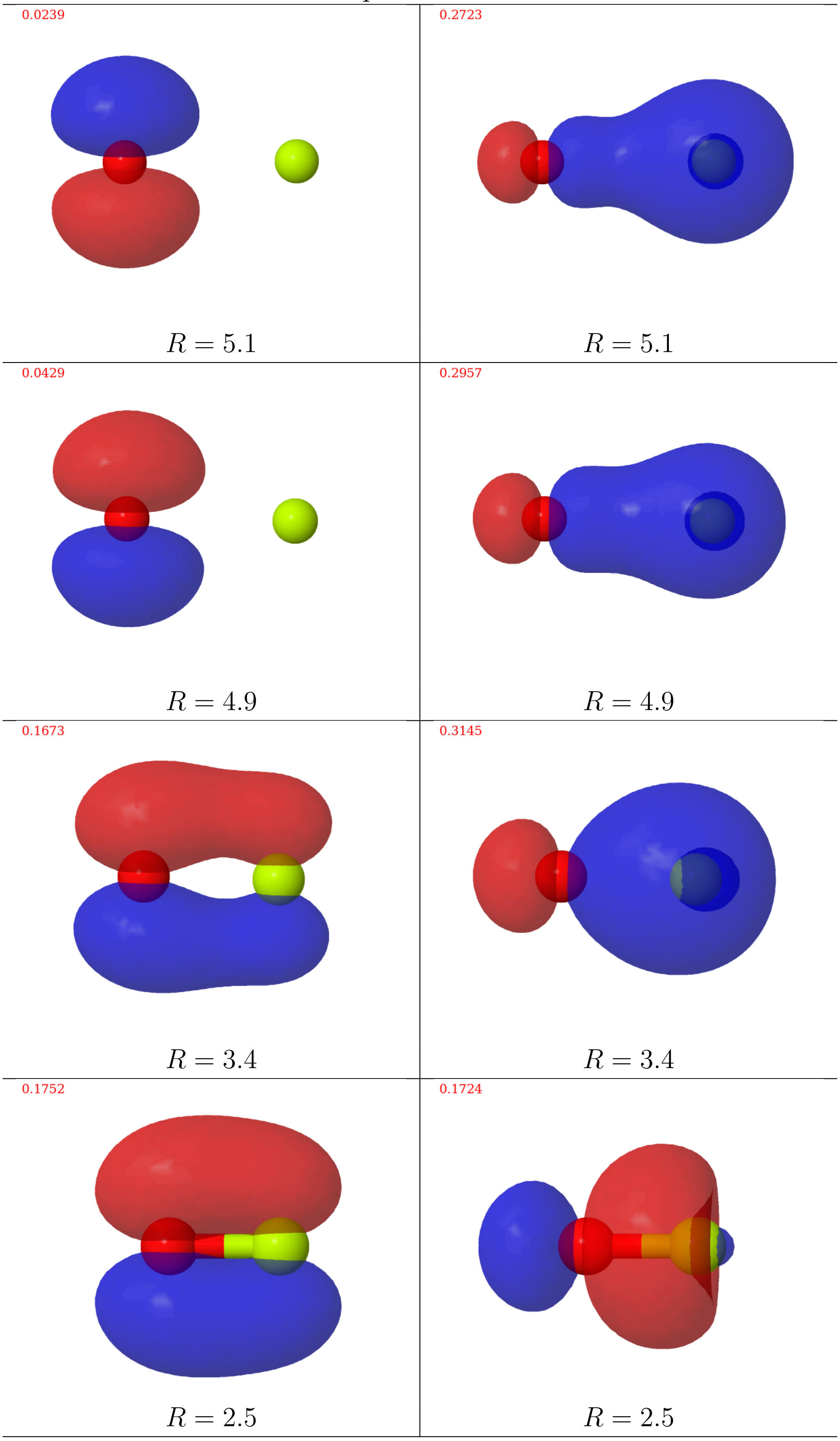

Supplementary References

[1] G. M. J. Barca, C. Bertoni, L. Carrington, D. Datta, N. De Silva, J. E. Deustua, D. G. Fedorov, J. R. Gour, A. O. Gunina, E. Guidez, T. Harville, S. Irle, J. Ivanic, K. Kowalski, S. S. Leang, H. Li, W. Li, J. J. Lutz, I. Magoulas, J. Mato, V. Mironov, H. Nakata, B. Q. Pham, P. Piecuch, D. Poole, S. R. Pruitt, A. P. Rendell, L. B. Roskop, K. Ruedenberg, T. Sattasathuchana, M. W. Schmidt, J. Shen, L. Slipchenko, M. Sosonkina, V. Sundriyal, A. Tiwari, J. L. Galvez Vallejo, B. Westheimer, M. Wloch, P. Xu, F. Zahariev and M. S. Gordon, *J. Chem. Phys.*, 2020, **152**, 154102.

[2] R. F. W. Bader, *Atoms in Molecules*, Oxford University Press, Oxford, 1990.

[3] A. D. Becke and K. E. Edgecombe, *J. Chem. Phys.*, 1990, **92**, 5397.

[4] M. A. Blanco, A. Martín Pendás and E. Francisco, *Journal of Chemical Theory and Computation*, 2005, **1**, 1096–1109.

[5] D. Menéndez-Crespo, A. Costales, E. Francisco and A. Martín Pendás, *Chem. Eur. J.*, 2018, http://dx.doi.org/10.1002/chem.201800979.

[6] A. Martín Pendás and E. Francisco, A QTAIM/IQA code (Available from the authors upon request).

[7] C. Outeiral, M. A. Vincent, Á. Martín Pendás and P. L. A. Popelier, *Chem. Sci.*, 2018, **9**, 5517–5529.

[8] E. Francisco, A. Martín Pendás and M. A. Blanco, *J. Chem. Phys.*, 2007, **126**, 094102–1–094102–13.

[9] A. Martín Pendás, E. Francisco and M. A. Blanco, *J. Chem. Phys.*, 2007, **127**, 144103.

[10] C. Outeiral, M. A. Vincent, A. Martín Pendás and P. L. A. Popelier, *Chem. Sci.*, 2018, **9**, 5517–5529.

[11] E. Francisco, A. Martín Pendás, M. García-Revilla and R. Á. Boto, *Comput. Theor. Chem.*, 2013, **1003**, 71 – 78.

[12] A. Martín Pendás, E. Francisco and M. A. Blanco, *Phys. Chem. Chem. Phys.*, 2007, **9**, 1087–1092.

[13] A. Martín Pendás and E. Francisco, *Phys. Chem. Chem. Phys.*, 2018, **20**, 16231–16237.

[14] M. Menéndez, R. Álvarez Boto, E. Francisco and Á. Martín Pendás, *J. Comp. Chem.*, 2015, **36**, 833–843.

[15] E. Francisco, A. Martín Pendás and M. Blanco, *Comput. Phys. Commun.*, 2008, **178**, 621 – 634.

[16] Y. W. Parr, Robert, *Density-functional theory of atoms and molecules*, Oxford University Press. Clarendon Press, New York, 1989.

[17] R. Ponec, *J. Math. Chem.*, 1998, **23**, 85–103.

[18] A. Martín Pendás and E. Francisco, *J. Chem. Theory Comput.*, 2018, **15**, 1079–1088.
