# Supplementary material for: The role of references and the elusive nature of the chemical bond
Source: Nat Commun. 2022 Jun 9;13:3327. doi: 10.1038/s41467-022-31036-6 (PMC9184482; doi:10.1038/s41467-022-31036-6)
Supplement: Supplementary file 3 — Description of Additional Supplementary Information [file 41467_2022_31036_MOESM3_ESM.docx]

**Supplementary Data 1**. CAS[8,8]/6-311G(p) IQA raw and EDF data in BeO. All data in a.u. A is Be, B is O. The IQA data show, in order, the kinetic energy of A, B, the self-energy of A, B, the interaction energy and its exchange-correlation and classical components, the topological charge of A, the localisation indices of A and B and the delocalization index.
