# Supplementary material for: The role of references and the elusive nature of the chemical bond
Source: Nat Commun. 2022 Jun 9;13:3327. doi: 10.1038/s41467-022-31036-6 (PMC9184482; doi:10.1038/s41467-022-31036-6)
Supplement: Supplementary file 4 — Supplementary Data 1 [file 41467_2022_31036_MOESM4_ESM.pdf]

IQA data

IQA atomic $T^{A,B}, E_{self}^{A,B}, E_{int}^{AB}, V_{xc}^{AB}, V_{cl}^{AB}, Q^{A}, \lambda^{A,B}, \delta^{AB}$ data, in a.u. A is Be, while B is the O atom.

#R	KinA	KinB	NetA	NetB	Interaction	XC	Classic	QA	QB	LocA	LocB	DI
0.25	14.517830	75.512494	-13.866948	-74.704803	-0.973900	-0.232767	-0.741133	1.409476	-1.406914	2.089351	8.904241	1.001288
2.4	14.395661	75.365656	-13.892854	-74.683654	-0.991725	-0.208406	-0.783319	1.422779	-1.419963	2.091993	8.933090	0.969287
2.48	14.350878	75.297986	-13.906954	-74.677726	-0.988318	-0.200249	-0.788069	1.417832	-1.416859	2.097936	8.932005	0.968114
2.5	14.340887	75.280857	-13.910289	-74.678100	-0.984659	-0.198073	-0.786586	1.416444	-1.414089	2.099464	8.928597	0.967231
2.6	14.299998	75.207135	-13.927541	-74.675654	-0.970173	-0.190318	-0.779855	1.404518	-1.403152	2.109307	8.916194	0.971767
2.75	14.261966	75.112666	-13.956341	-74.680005	-0.929536	-0.183099	-0.746437	1.370374	-1.370357	2.132273	8.872988	0.994704
3.0	14.241832	74.990691	-14.021051	-74.704356	-0.816513	-0.178834	-0.637679	1.269752	-1.269148	2.200102	8.738727	1.059962
3.2	14.246488	74.924690	-14.080876	-74.725600	-0.712554	-0.175669	-0.536885	1.167940	-1.168091	2.281251	8.617313	1.101739
3.4	14.259842	74.880937	-14.142001	-74.745715	-0.607818	-0.168245	-0.439573	1.062210	-1.062088	2.384043	8.508281	1.107431
3.5	14.268400	74.866339	-14.172107	-74.755153	-0.556910	-0.162350	-0.394560	1.010307	-1.010450	2.443354	8.464106	1.092826
3.6	14.277342	74.856458	-14.201190	-74.764293	-0.507570	-0.154550	-0.353020	0.961401	-0.961704	2.507021	8.430127	1.063458
3.8	14.294243	74.855429	-14.254698	-74.783293	-0.414128	-0.128162	-0.285966	0.883604	-0.883463	2.654879	8.421893	0.922946
4.0	14.300607	74.862346	-14.287275	-74.796837	-0.349806	-0.102708	-0.247098	0.844464	-0.844644	2.772661	8.461746	0.765953
4.5	14.321328	74.854853	-14.344641	-74.811961	-0.238074	-0.081535	-0.156539	0.744315	-0.744388	2.931125	8.419818	0.649203
4.6	14.329175	74.852230	-14.356727	-74.812748	-0.218447	-0.079994	-0.138453	0.717508	-0.717697	2.960381	8.395577	0.644419
4.8	14.356187	74.842845	-14.386092	-74.811249	-0.178246	-0.078847	-0.099399	0.639577	-0.639415	3.032412	8.311367	0.655896
4.85	14.371347	74.837585	-14.399259	-74.810062	-0.163599	-0.079189	-0.084410	0.598999	-0.599058	3.067445	8.265463	0.667209
4.9	14.397026	74.829223	-14.419947	-74.808089	-0.142432	-0.078656	-0.063776	0.531876	-0.531828	3.131404	8.195075	0.673425
5.0	14.434297	74.817824	-14.450196	-74.804120	-0.112125	-0.072657	-0.039468	0.432048	-0.432000	3.249744	8.113762	0.636398
5.1	14.457616	74.810348	-14.469836	-74.800796	-0.092589	-0.065665	-0.026924	0.366251	-0.366173	3.340931	8.073332	0.585580
5.25	14.479953	74.804835	-14.491273	-74.793972	-0.074131	-0.056301	-0.017830	0.301222	-0.301230	3.441478	8.043886	0.514652
5.4	14.502010	74.795199	-14.508342	-74.792386	-0.055744	-0.046451	-0.009293	0.233490	-0.233542	3.549987	8.016957	0.433160
5.5	14.511465	74.792357	-14.517231	-74.789724	-0.047974	-0.041172	-0.006802	0.204099	-0.203956	3.601309	8.009382	0.389022
5.75	14.531233	74.785330	-14.534367	-74.785484	-0.032232	-0.029716	-0.002516	0.140677	-0.140617	3.714890	7.996156	0.288834
6.0	14.538595	74.786555	-14.547411	-74.778095	-0.024714	-0.023080	-0.001634	0.112388	-0.112343	3.773515	7.998217	0.228179
6.5	14.552824	74.782074	-14.561108	-74.774708	-0.012452	-0.012394	-0.000058	0.060345	-0.060241	3.875701	7.996302	0.127788
7.0	14.559712	74.779907	-14.567226	-74.773641	-0.006552	-0.006767	0.000215	0.033591	-0.033527	3.930111	7.997217	0.072545
7.5	14.565210	74.776455	-14.570941	-74.773805	-0.003035	-0.003261	0.000226	0.015775	-0.015723	3.966680	7.998156	0.035060
8.0	14.566200	74.775930	-14.571595	-74.774370	-0.001736	-0.001896	0.000160	0.009683	-0.009640	3.979611	7.998906	0.021397
8.5	14.566873	74.775566	-14.571874	-74.774802	-0.000962	-0.001093	0.000131	0.005920	-0.005853	3.987579	7.999345	0.012941
9.0	14.567340	74.775325	-14.571972	-74.775113	-0.000500	-0.000624	0.000124	0.003562	-0.003506	3.992548	7.999600	0.007739

EDFs

Distances in a.u. Probability of finding n_{α}, n_{β} in the Be,O basins.

#R	(2244)	(3135) Triplet	(2145)	(3234)	(1155)
2.5	0.0498599050537983	0.0206788932676367	0.1902666936870863	0.0032669623264813	0.5114080413324117
2.75	0.0550754201545226	0.0237798339484219	0.2007334209810956	0.0034546093356226	0.4825147253896432
3.0	0.0697698378468090	0.0304472970684523	0.2182787309798007	0.0044752648938809	0.4180272941987436
3.2	0.0870156235818956	0.0372891309832493	0.2336584057267061	0.0056554986010151	0.3551923180645408
3.4	0.1073050209073973	0.0439964088648517	0.2480680925245348	0.0068929453365141	0.2901535014412136
3.5	0.1181952867330878	0.0468172206735710	0.2554908160303042	0.0074805986721183	0.2579772425637937
3.6	0.1289764937162451	0.0487504178112618	0.2636742260397542	0.0079980768194520	0.2262644704217984
3.8	0.1452659166032712	0.0457688143922971	0.2922139310793535	0.0082836036157677	0.1588382524343160
4.0	0.1539606006310572	0.0371698103383768	0.3232907996141784	0.0077886640451796	0.1076285675462599
4.2	0.1706097988210530	0.0316782587686273	0.3345565871569195	0.0079188026301208	0.0795050758142855
4.4	0.1984132191272018	0.0265838589451098	0.3362859605200222	0.0084096732126413	0.0577376086757803
4.5	0.2188293986179977	0.0238866830382408	0.3334174063666861	0.0087691376225537	0.0481388029684239
4.6	0.2433547556464890	0.0208438946560433	0.3279429914476268	0.0090051973874566	0.0404489676009715
4.8	0.3246846607799641	0.0135332686363361	0.2999079787056971	0.0092586370159076	0.0273721272374395
4.85	0.3682797579227776	0.0110198368709294	0.2817273006313503	0.0092124379478307	0.0293612312938913
4.9	0.4403002169439753	0.0078135145662849	0.2502550488959280	0.0091108653878997	0.0250066851739607
5.0	0.5453947820101188	0.0044115274500250	0.2047858892882857	0.0082079173053833	0.0195771897749176
5.1	0.6131573159930899	0.0029218830316681	0.1753820972963679	0.0074052399690865	0.0152273413346520
5.25	0.6834411648871525	0.0016407482620516	0.1449924853745038	0.0058477314617740	0.0115402837132305
5.4	0.7482774675879856	0.0011258635038084	0.1154221862402070	0.0057360056557480	0.0071480606788505
5.5	0.7793539597310989	0.0008303825734748	0.1014285704835429	0.0051110021340411	0.0057186525992024
5.75	0.8439456751472555	0.0004394108335972	0.0717003033745004	0.0043435672114708	0.0029979967706013
6.0	0.8795851623360246	0.0001813903600478	0.0564277725086993	0.0024581818889122	0.0022267245515862
6.5	0.9345161040942057	0.0000514311293665	0.0308297696785414	0.0014339742540332	0.0007464541373424
7.0	0.9632848832922027	0.0000156316672808	0.0173524545485315	0.0008281724549512	0.0002581476599878
7.5	0.9823744052385484	0.0000038404662044	0.0082786598801658	0.0004689819772228	0.0000702918320814
8.0	0.9892412871240663	0.0000013303687854	0.0050774263657422	0.0002659301176206	0.0000261843057996
8.5	0.9934555940956586	0.0000004518678161	0.0030842926923366	0.0001489144732580	0.0000096039109551
9.0	0.9960716403335974	0.0000001513759155	0.0018507772653781	0.0000831821964061	0.0000034452386691
